# Metagenomic Analyses Reveal Gut Microbial Profiles of *Cnaphalocrocis medinalis* Driven by the Infection of Baculovirus CnmeGV

**DOI:** 10.3390/microorganisms12040757

**Published:** 2024-04-09

**Authors:** Chuanming Li, Guangjie Han, Lixin Huang, Yurong Lu, Yang Xia, Nan Zhang, Qin Liu, Jian Xu

**Affiliations:** National Experimental Station of Yangzhou for Agricultural Microbiology, Jiangsu Lixiahe Institute of Agricultural Sciences, Yangzhou 225008, China; liming0595@163.com (C.L.); hanguangjie177@163.com (G.H.); huanglx0514@126.com (L.H.); lyrwf@163.com (Y.L.); xiayeung@163.com (Y.X.); znfezhangnan@hotmail.com (N.Z.); bio-lq@126.com (Q.L.)

**Keywords:** metagenomic, baculovirus, CnmeGV, gut microbial, *Cnaphalocrocis medinalis*

## Abstract

The composition of microbiota in the digestive tract gut is essential for insect physiology, homeostasis, and pathogen infection. Little is known about the interactions between microbiota load and oral infection with baculoviruses. CnmeGV is an obligative baculovirus to *Cnaphalocrocis medinalis*. We investigated the impact of CnmeGV infection on the structure of intestinal microbes of *C. medinalis* during the initial infection stage. The results revealed that the gut microbiota profiles were dynamically driven by pathogen infection of CnmeGV. The numbers of all the OTU counts were relatively higher at the early and later stages, while the microbial diversity significantly increased early but dropped sharply following the infection. The compositional abundance of domain bacteria Firmicutes developed substantially higher. The significantly enriched and depleted species can be divided into four groups at the species level. Fifteen of these species were ultimately predicted as the biomarkers of CnmeGV infection. CnmeGV infection induces significant enrichment of alterations in functional genes related to metabolism and the immune system, encompassing processes such as carbohydrate, amino acid, cofactor, and vitamin metabolism. Finally, the study may provide an in-depth analysis of the relationship between host microbiota, baculovirus infection, and pest control of *C. medinalis*.

## 1. Introduction

The insect gut is a life-sustaining organ, where the organisms interact most actively with the external ecosystem to maintain their physiological and immunological homeostasis [1]. In contrast to other insect organs, the gut is exposed to a constant flow of the microbial environment throughout the insect’s life [2]. Among the various microbes in the insect gut, including bacteria, fungi, archaea, and protozoa, some are adapted to the gut environment, harbor and proliferate in the gut ecosystem, and constitute the gut microbiota, while some other microbes, such as infectious pathogens, are harmful, causing host pathology or even death [3]. Mutualistic relationships between gut bacteria and arthropods are common in nature [2]. The roles of symbionts in these associations with insects are remarkably diverse, including in nutrition, physiology, and behavior [4]. The resident microorganisms can also protect their insect hosts against pathogens and other natural enemies [5,6,7,8]. The normal gut symbionts form a stable community that resists the invasion of non-native bacteria and the expansion of pathobionts [9]. Following the discovery that symbiotic microorganisms profoundly shape mammalian immunity [10], increasing studies have found evidence that commensal microbiota present in the gut-modulated insect immune responses [11,12,13] provide protection against pathogens [14,15] or promote pathogen infection [13,16]. The mechanism of interaction between gut microbiota and infection pathogens is not fully understood, which will be a fascinating field of research.

Insect gut microbiota was dynamically changed in type and proportion and was observed under different habitat conditions, insect diets, and at different developmental stages [17,18]. Herbivorous insects ingest microorganisms that are present in plants [19]. Food consumption was considered a major exogenous factor that directly influenced the composition of insect gut microbiota [20]. Gut bacterial diversity was significantly higher in omnivorous insects than in stenophagous insects [18]. Some studies also suggested that the gut microbiota was essential in the insect response to infectious pathogens. Midgut bacteria were required for *Bacillus thuringiensis* (Bt) insecticidal activity [21]. Baculoviruses are natural enemies of agricultural and forest insect pests. They play an important role in biological pest control [22], and these viruses are among the most typical and well-studied viruses in the interaction research of pathogens with insects [23]. Infection with the baculovirus SeMNPV significantly increased the intestinal bacterial loads in *Spodoptera exigua* Hübner, 1808 (Lepidoptera: Noctuidae) larvae, with approximately 100-fold higher counts for *Enterococcus* [24]. Silkworm intestinal flora diversity changed significantly after bidensovirus BmCPV infection. The number of bacterial species in silkworms decreased at all classification levels post infection, where the abundances of *Enterococcus* and *Staphylococcus* increased and *Delftia* decreased [25]. The increases in the abundances of *Anderseniella*, *Simplicispira*, and *Enterococcus* in silkworms were associated with BmBDV infection [26]. Although existing research suggests that infectious pathogens induce an imbalance in the gut microbiota, these interactions have not yet been extensively studied but have been demonstrated in several associations.

The rice leaf folder, *Cnaphalocrocis medinalis* Güenée, 1854 (Lepidoptera: Pyralidae), is an oligophagous pest that feeds almost exclusively on rice leaves and causes severe damage to rice in Asia. The larva of *C. medinalis* hosts a stable and diverse microorganism community in the midgut [27], with dominant core residents of *Enterococcus* species independent of the changes in diets [28]. The *C. medinalis* granulovirus (CnmeGV) is an obligative baculovirus to the pest and might be developed as an effective microbial biopesticide against *C. medinalis* [29]. CnmeGV infection begins after ingestion of virus-contaminated food [30]. The infection of CnmeGV has been shown to induce a systemic antiviral immune response in *C. medinalis*, including the RNAi and oxidative stress antiviral mechanisms [31]. The immune response of *C. medinalis* may display some effects on the gut microbiota community, thereby impacting the viral infection. Based on this hypothesis, the current study has been focused on the interaction between gut microbiota and baculovirus infection, further to characterize the gut microbiota in *C. medinalis* infected with CnmeGV in the initial infection stage by comparing the gut bacterial communities of healthy *C. medinalis* larvae with those affected by CnmeGV. We also aimed to evaluate dynamic changes in the gut microbiota of CnmeGV-infected *C. medinalis*.

## 2. Materials and Methods

### 2.1. Insect and Virus

The *C. medinalis* were raised on maize seedlings in our laboratory as previously described [32]. The colony was kept for years under standard conditions of a photoperiod of 14 h:10 h (light:dark) and relative humidity of 70.0%, at 25 ± 1 °C. The qRT-PCR [33] was performed before the experiments to ensure that the colony was negative for CnmeGV.

The CnmeGV was isolated from the carcasses of *C. medinalis* and multiplied in the laboratory using living larvae following a previous study [29]. The infectious bodies were collected and homogenized in distilled water. Second-instar larvae were constantly fed with virus-added artificial food until the individuals showed obvious symptoms of GV infection. Final purification of the virus was carried out with sucrose density gradient centrifugation procedures [34], and the concentration was quantified to 1 × 10^6^ OB (occlusion body)·mL^−1^ by dark-field microscopy using 0.02 mm depth cytometers.

### 2.2. Oral Infection and Midgut Collection

Oral infection was performed with the leaf-dipping method. Fresh corn seedling leaves were cut into 1 × 1 cm squares and soaked in CnmeGV at a concentration of 1 × 10^6^ OB·mL^−1^ for 30 s. Individual leaf pieces were placed in sterilized 2 mL centrifuge tubes that were left open until the surface was dry. Two-day-old third-instar larvae were starved for 4 h and then orally infected with CnmeGV by feeding in the centrifuge tubes described above. The addition of fresh and untreated leaves was continued after the GV-soaked leaves were eaten up. The mock-infected group received pieces of leaf washed with sterile distilled water. Midgut samples were collected as described in previous study [28] at 24 h, 48 h, and 72 h post-infection (hpi). Ten larvae were collected for one biological replication, and five replicates were collected for each treatment.

### 2.3. Metagenomic Sequencing and Assembly

Total metagenomic DNA was extracted using QIAampDNA Stool Mini Kit (Qiagen, Hilden, Germany). The total DNA sequencing was carried out through Microeco Tech Co., Shenzhen, China. Metagenomic sequencing and data preprocessing methods were the same as previously described [28]. We selected the paired-end (PE) library with a 350 bp insert size. All the samples were sequenced using the PE150 (2 × 150) protocol on the Illumina Novaseq 6000 (Illumina, San Diego, CA, USA).

Trimmomatic (v0.39) was used to remove the subsequent sequence based on the average quality score lower than 20 of the reads < 50 bp [35]. Clean data were obtained from raw data through the removal of the host sequencing using Bowtie2 (v2.4.5) [36]. FastQC (v0.11.9) was used for quality control of the sequencing data. The high-quality sequences were assembled through MEGAHIT(v1.2.9) [37], and the genes were predicated using prodigal (v2.6.3) [38]. CD-hit (v4.8.1) removed redundant genes to obtain the non-redundant gene catalog [39]. The clean data were mapped to the non-redundant gene catalog by employing Salmon (v1.6.0) to calculate the value of reads per kilobase per million (RPKM) [40]. The reads were aligned using Kraken2 (v2.1.1) for functional taxonomy assignment and taxonomic identity [41]. Predicted unigenes were annotated using eggNOG-mapper (v2.1.7) (based on DIAMOND) that will perform mapping to the Kyoto Encyclopedia of Genes and Genomes (KEGG) databases [42].

### 2.4. Data Analysis

The interaction circle diagram of species composition was drawn by Krona [43]. Alpha-diversity indexes, including the Chao 1, Shannon, and Simpson, were calculated in QIIME 2 [44], and the statistical significance was calculated using the two-tailed, unpaired Student’s *t*-test, followed by the Benjamini, Krieger and Yekutieli procedure for false discovery rate (FDR) correction. We used the Non-Metric Multi-Dimensional Scaling (NMDS) method to evaluate the difference in beta diversity between treatments [45]. LefSe (linear discriminant analysis (LDA) effect size) was performed using the online Galaxy workflow framework to identify features with significant differences between treatment groups. The Wilcoxon rank-sum test tested the significance of the difference, and the *p* value was corrected as FDR with the Benjamini–Hochberg method (http://huttenhower.sph.harvard.edu/galaxy/, accessed on 24 May 2022). The random forest model was used to generate a heatmap of important taxa [46]. The differential abundance of functional modules between groups was drawn by the Statistical Analysis of Metagenomic Profiles (STAMP).

## 3. Results

### 3.1. Alterations of Gut Microbiota Composition in Cnmegv-Infected C. medinalis

Metagenomic sequencing produced 1.5 × 10^8^ clean reads in total that passed the quality control, and the numbers of clean reads ranged from 2.48 × 10^7^ to 2.72 × 10^7^ in each sample (Supplementary Table S1). High sampling coverage was achieved in all samples according to rarefaction curves generated from the Operational Taxonomic Units (OTUs) (Supplementary Figure S1). Moreover, bacteria, fungi, archaea, and phages were also reported in all samples. Bacteria accounted for 99.5% of total gut symbionts, while fungi only accounted for 0.5%, and archaea and phage proportions were low (Supplementary Table S2). Only one OTU classified as a virus was identified as CnmeGV, which was present in all CnmeGV-infected samples but was absent in the mock-infected samples (Supplementary Table S3).

**Figure 1:**
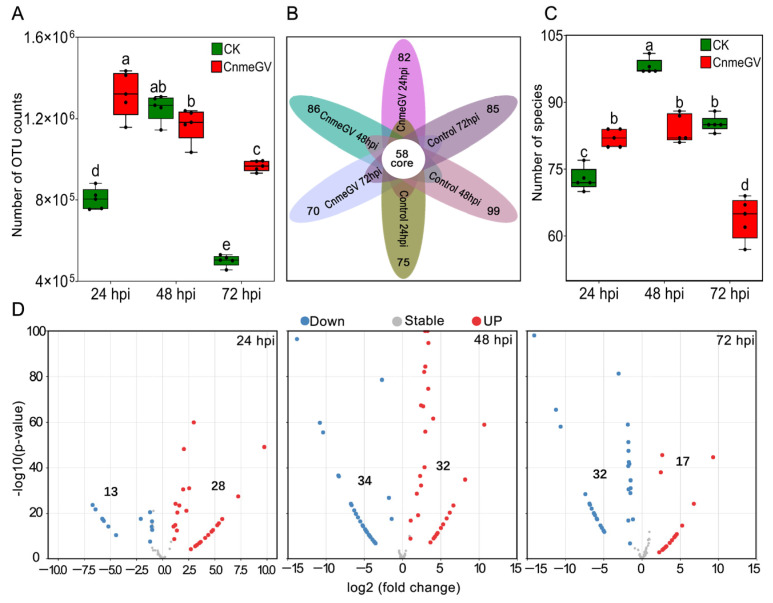
Significant changes in the species and abundance of gut microbiota after CnmeGV infection. (**A**) Box plot of the number of OUT counts. Box plots show high, low, and median values, with the lower and upper edges of each box denoting first and third quartiles, respectively. The dots represent biological replicates, *n* = 5, with a pool of 10 gut regions in each replicate. Multiple comparisons were performed with one-way ANOVA (Tukey’s post hoc test); different letters indicate significant differences at a *p*-value < 0.01; the same below. (**B**) Venn diagram of the observed OTUs. (**C**) Difference analysis of the observed species. (**D**) MA plot of enrichment and depletion species obtained by pairwise comparison of CnmeGV-infected group vs. mock-infected group.

The OTUs of both treatment groups were dynamically changed following the detection time but showed quite a difference between the treatments (F _(5, 24)_ = 105.7, *p* < 0.01). The OTU counts of the CnmeGV-infected group were significantly higher than those of the mock-infected group at 24 (*p* < 0.01, *t* _(24)_ = 12.07) and 72 hpi (*p* < 0.01, *t* _(24)_ = 10.86), while being similar at 48 hpi (*p* = 0.97, *t* _(24)_ = 1.94) (Figure 1A). According to the OTUs, 129 gut bacteria were identified from all the treatments at the species level. The Venn diagram showed that 70~99 species were contained in each group, and 58 species were shared by all the treatment groups (Figure 1B). The number of species of bacteria in the virus-infected group had a marked increase at 24 hpi (*p* < 0.01, *t* _(24)_ = 5.07) but significantly decreased at 48 (*p* < 0.01, *t* _(24)_ = 7.72) and 72 hpi (*p* < 0.01, *t* _(24)_ = 11.68), compared with the control (F _(5, 24)_ = 81, *p* < 0.01) (Figure 1C). 

Using OTU counts from the mock-infected group as a control and an adjusted *p*-value cutoff of 0.01, we conducted the MA plot to show the number of species with significant changes in abundance. There were significantly more enriched species at 24 hpi (28 vs. 13), and significantly more depleted species at 72 hpi (32 vs. 17), while enriched species were almost equal to the depleted species at 48 hpi (32 vs. 34). The highest number of significantly changed species was at 48 hpi, with 66 species demonstrating significant changes (Figure 1D).

**Figure 2:**
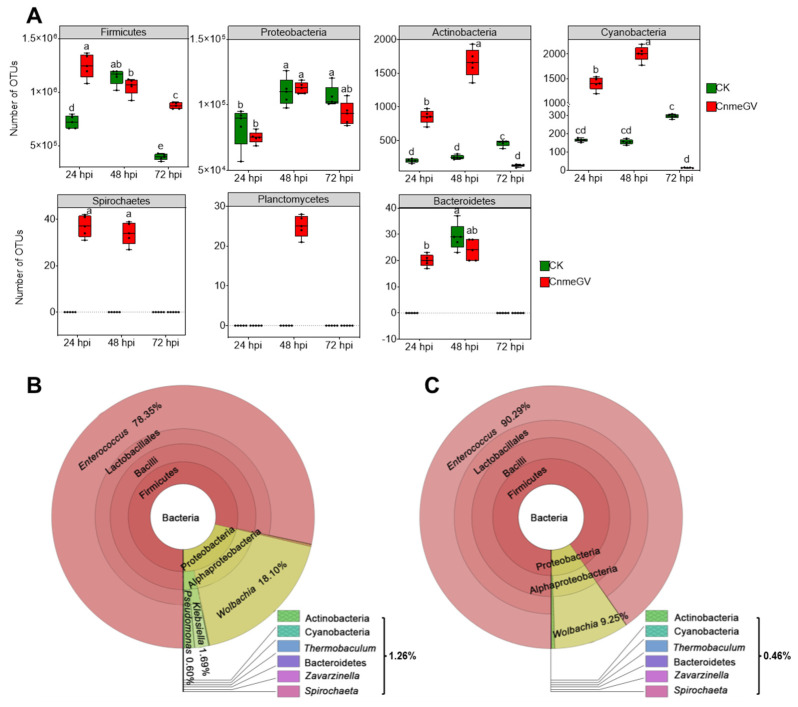
Absolute and relative abundance of bacteria in the larvae gut of *C. medinalis* after CnmeGV infection. (**A**) Box plots of OTU counts for the gut microbiota at the phylum level. Multiple comparisons were performed with one-way ANOVA (Tukey’s post hoc test); different letters indicate significant differences at a *p*-value < 0.01. (**B**,**C**) Relative abundance at different taxonomic terms annotated using Krona ((**B**) CnmeGV-infected at 72 hpi, (**C**) mock-infected at 72 hpi; the rest are in Supplementary Figure S3). Different classification levels are represented by inner and outer circles, with areas of different colors being proportional to the relative abundance. The following website also has dynamic and more detailed information about Krona (accessed on 14 June 2022): https://licm.github.io/kronacnmeGV.github.io/Krona1.html.

There were significant changes of gut microbiota at the phylum level based on the relative abundance (*p* < 0.01) (Supplementary Figure S2). The OTU counts of Firmicutes, Actinobacteria, Cyanobacteria, Spirochaetes, Planctomycetes, and Bacteroidetes were all higher in the CnmeGV-infected group than the mock-infected group at 24 hpi (*p* < 0.01). Furthermore, Actinobacteria, Cyanobacteria, Spirochaetes and Planctomycetes were all higher in the CnmeGV-infected group than the mock-infected group at 48 hpi (*p* < 0.01). The Firmicutes were higher with Actinobacteria, and Cyanobacteria were lower at 72 hpi (*p* < 0.01). The OTU counts of Proteobacteria showed there were no significant changes between the infected and mock-infected group at each detecting time (Figure 2A). The dominant bacterial communities were similar between infected and mock-infected groups. Firmicutes (79.2−94.4%) and Proteobacteria (6.5−21.0%) were the dominant bacteria at the phylum level, Enterococcaceae (78.1−94.3%) and Anaplasmataceae (5.2−18.3%) at the family level, and *Enterococcus* (78.4−93.7%) and *Wolbachia* (5.3−18.2%) at the genus level (Figure 2B,C; Supplementary Figure S3).

### 3.2. Alterations of Gut Microbiota Diversity in CnmeGV-Infected C. medinalis

**Figure 3:**
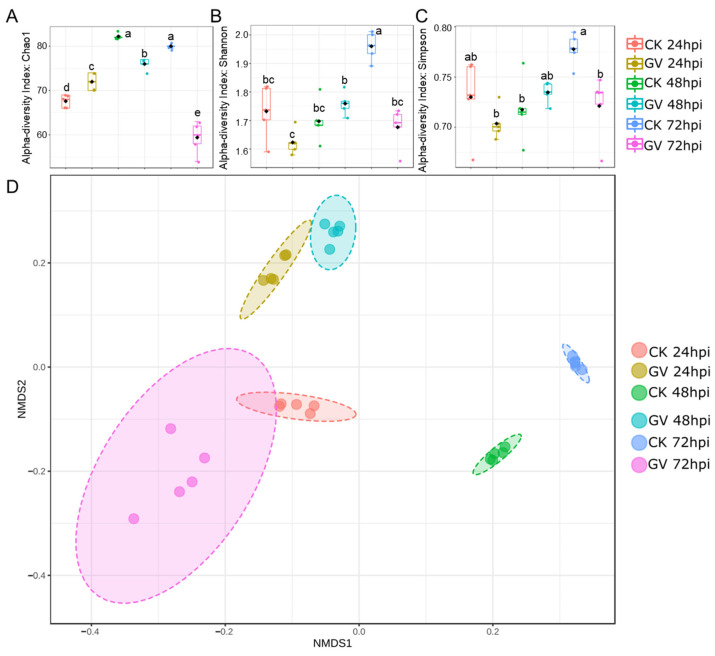
Alterations of gut microbiota diversity in CnmeGV-infected *C. medinalis*. (**A**–**C**) Box plot of alpha-diversity indexes, measured using Chao 1(**A**), Shannon (**B**) and Simpson (**C**). Multiple comparisons were performed with one-way ANOVA (Tukey’s post hoc test); different letters indicate significant differences at a *p*-value < 0.01. (**D**) The non-metric multidimensional scaling (NMDS) plots of beta diversity.

To assess the temporal dynamics of gut microbiota, we quantified alpha and beta diversity at the species level. The community richness of gut microbiota composition between CnmeGV-infected and mock-infected groups showed a considerable difference in values measured by the Chao 1 index (*p* < 0.01, F_(5, 24)_ = 100.7). Compared with the mock-infected group, the index of the infection group changed from slightly higher at 24 hpi (*p* < 0.01, t = 3.920, df = 8) to sharply lower at 72 hpi (*p* < 0.01, t = 12.63, df = 8) (Figure 3A). The Shannon and Simpson indexes were used to calculate the similarity and diversity of the two groups. The variation tendencies of the Shannon index and the Simpson index were similar, but differed between infected and mock-infected groups, respectively. The two indexes of the infection group were significantly lower than those of the non-infection group at 72 hpi (*p* < 0.01, t = 7.404, df = 8; *p* < 0.01, t = 3.564, df = 8) (Figure 3B,C).

Separations among treatments were visualized through NMDS of a Bray–Curtis dissimilarity matrix using square transformation and Wisconsin standardization. The beta diversity of infected and mock-infected groups showed a significant difference according to the NMDS coordinates (stress = 0.018, F = 51.19, *p* < 0.01). The mock-infected group was scored as having low NMDS1 and NMDS2 values at 24 hpi, and the NMDS1 value then increased following the detection time to much higher values at 48 and 72 hpi. The CnmeGV-infected group showed high NMDS2 and low NMDS1 values at 24 and 48 hpi, respectively, and then the group was scored as having low NMDS1 and NMDS2 values at 72 hpi, much closer to the 24 hpi mock-infected group (Figure 3D).

### 3.3. Gut Bacteria Associated with CnmeGV Infection

**Figure 4:**
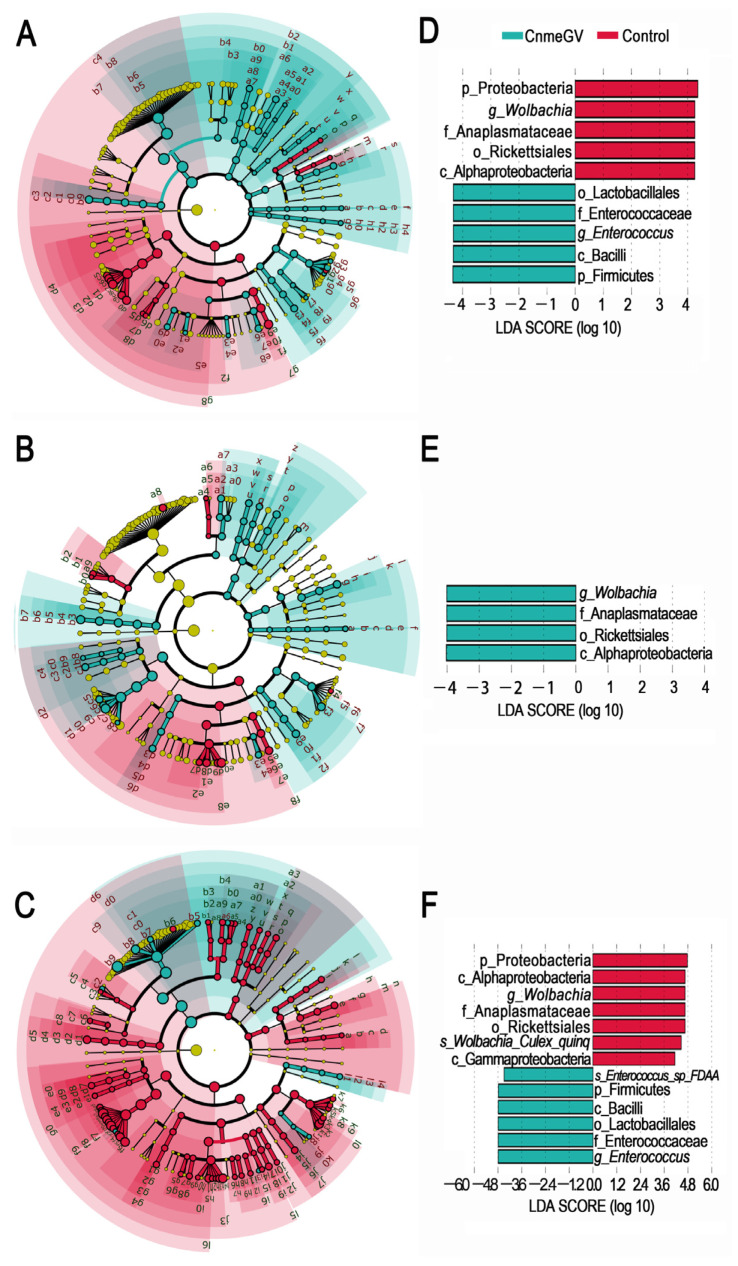
LEfSe analysis identifying taxonomic differences in the gut microbiota of the *C. medinalis* responding to CnmeGV-infection. (**A**–**C**) Cladogram plot showing the taxonomic groups that explain the most variation among the bacterial communities. Differences are represented in the color of the significantly enriched class (red indicating mock-infected, blue indicating CnmeGV-infected, yellow non-significant). (**A**) 24 hpi, (**B**) 48 hpi, (**C**) 72 hpi. The taxonomic names represented by the letters are presented in Supplementary Figure S5. (**D**–**F**) LDA score plot from LEfSe analysis indicating differentially enriched bacterial taxa associated either with CnmeGV-infection (blue) or mock-infection (red). (**D**) 24 hpi, (**E**) 48 hpi, (**F**) 72 hpi.

The LEfSe analysis was used to investigate the changes among the microbial profiles during the process of CnmeGV infection. The number of species with significant differences (LDA score higher than 2) was higher in the CnmeGV-infected group at 24 and 48 hpi (74 vs. 28 at 24 hpi; 65 vs. 20 at 48 hpi), and lower at 72 hpi (20 vs. 123) (Figure 4A–C, Supplementary Figures S4 and S5). A total of 10 gut bacterial clades were detected showing significant differences, with a LDA score higher than 4 at 24 hpi. *Wolbachia* was the most enriched biomarker in the mock-infected group, while *Enterococcus* was the most enriched biomarker in the CnmeGV-infected group (Figure 4D). On the contrary, *Wolbachia* was significantly enriched in CnmeGV-infected group at 48 hpi (Figure 4E). The 13 clades changed at 72 hpi with a LDA score higher than 4, where *Wolbachia* was the most enriched biomarker in the mock-infected group, and *Enterococcus* was the most enriched biomarker in the CnmeGV-infected group (Figure 4F).

**Figure 5:**
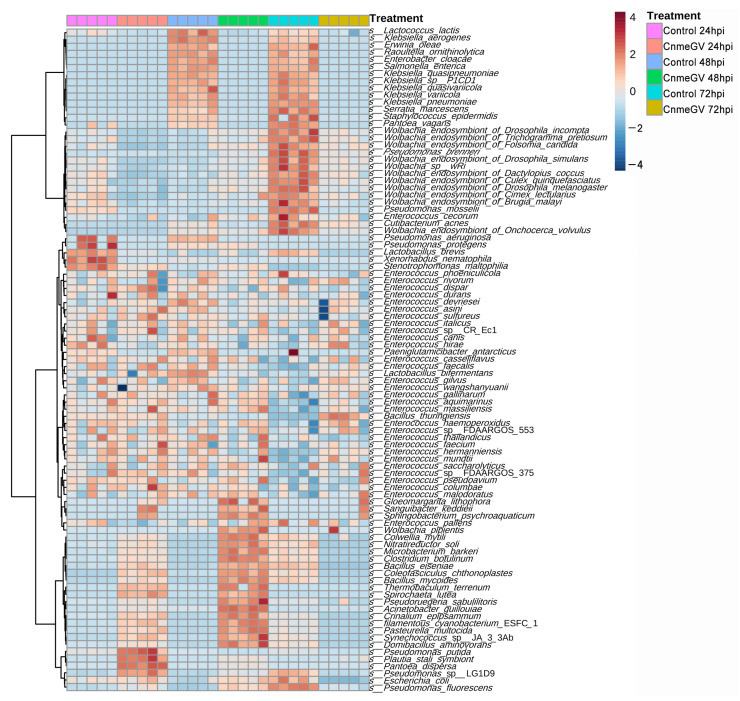
Heat map of the identified bacterial species’ relative abundances. Bacterial species were sorted based on Euclidean distance correlation with complete linkage.

We then compared the bacterial profile at the species level based on the relative abundance of all annotated species in infected and mock-infected groups. The differentially abundant microbes were divided into four groups (Figure 5): (1) Bacteria in the CnmeGV-infected group with increased abundance at 24, 48 hpi and decreased at 72 hpi. The *Pseudomonas putida*, *Plautia stali symbiont*, and *Pantoea dispersa* were the top three enriched species at 24 hpi. *Domibacillus aminovorans*, *Synechococcus* sp. JA33Ab, and *Pseudoruegeria sabulilitoris* were the top three enriched species at 48 hpi. All these species in this group exhibited lower abundance at 72 hpi. (2) Bacteria with substantially changed abundance but having no noteworthy association with different treatment groups. A total of 42 species were in this group, most classified as *Enterococcus*. (3) Bacteria with increased abundance only in the mock-infected group at 72 hpi. Most of the members in this group are *Wolbachia*. (4) Bacteria with increased abundance in the mock-infected group at both 48 and 72 hpi. This group contained 14 species, of which 6 species came from *Wolbachia*.

**Figure 6:**
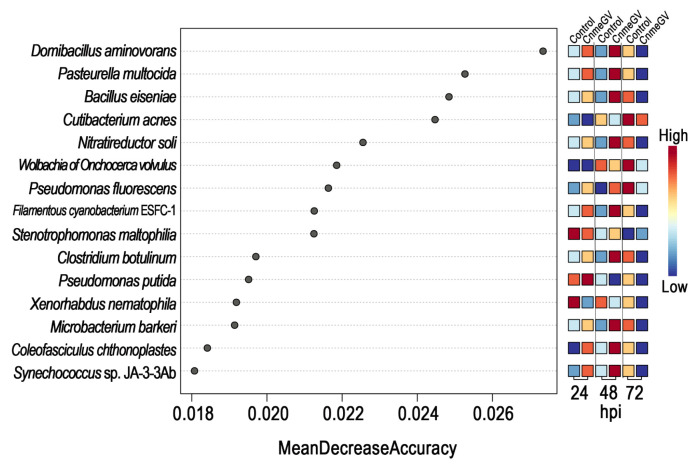
Variable importance according to the mean decrease in accuracy of the random forest classifier. Features are ranked by their contributions to classification accuracy (Mean Decrease Accuracy).

**Figure 7:**
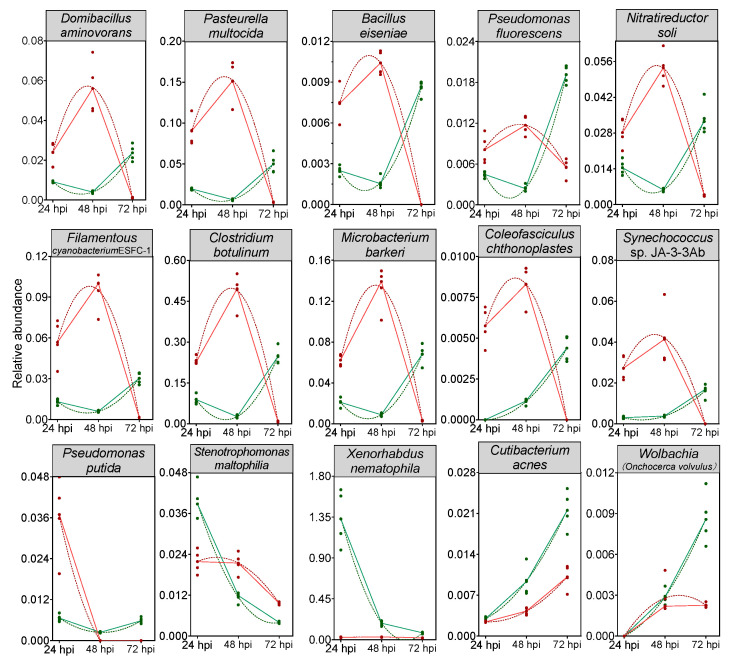
Trends of the abundance changes of the top 15 important bacteria species according to the random forest model. Red lines represent the CnmeGV-infected group, green lines represent the mock-infected group. The dots represent biological replicates (*n* = 5). Peak times of a second order polynomial (quadratic) fitting to the data as calculated by Graphpad v8.0.2 are shown (dashed curves).

A random forest model was then constructed to predict the biomarkers at species level and to test whether potential biomarkers can be used to represent CnmeGV infection status in *C. medinalis*. The area under the curve (AUC) was 91.4%, with a 95.0% confidence interval (CI) of 85.8% to 97.0%, suggesting that the prediction model possessed a huge discriminatory power for predicting the status of CnmeGV infection (Supplementary Figure S6). The contributions of the Top15 species are listed in Figure 6, where *Domibacillus aminovorans*, *Pasteurella multocida*, *Bacillus eiseniae* and *Cutibacterium acnes* contributed most to the identification of the infected and mock-infected groups. Out of the 15 species, 11 were classified in group 1 as described above: bacteria with increased abundance at 24, 48 hpi and decreased abundance at 72 hpi in the CnmeGV-infected group. In addition, we analyzed the variation trends of the abundance of all 15 species to further investigate the in-detail effect of CnmeGV infection on the gut microbiota (Figure 7). The results were consistent with the heat map and random forest model. These findings further confirmed that CnmeGV infection changed the abundance of specific bacteria, especially those in group 1.

### 3.4. Disrupted Bacteria Functions in CnmeGV-Infected Group

**Figure 8:**
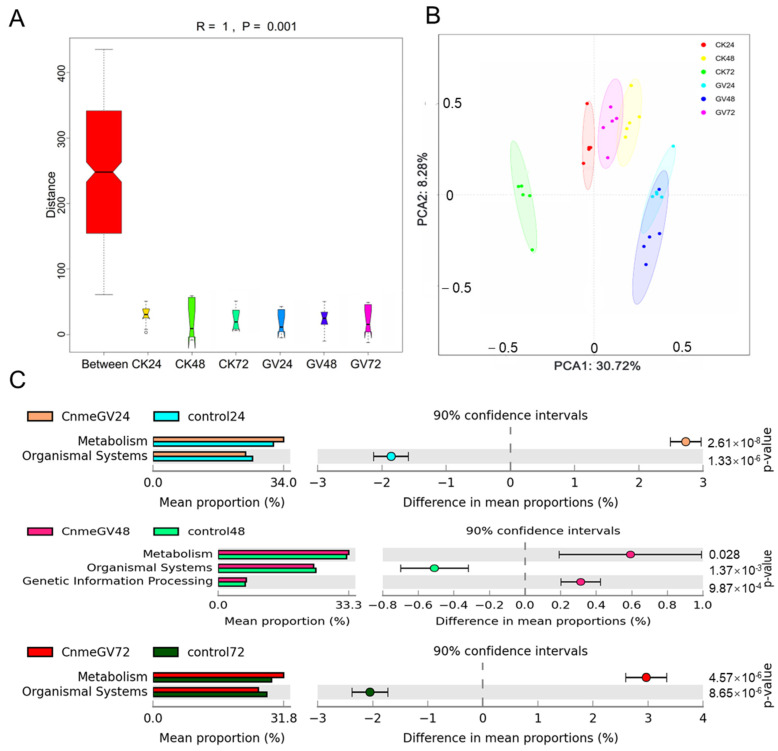
Microbial gene function annotation on KEGG in CnmeGV-infected and non-infected groups. (**A**) ANOSIM analyses of the predicated KEGG orthologous (KO) modules among different groups. (**B**) PCoA analyses of all the predicated KEGG modules according to the Bray–Curtis distances. (**C**) Comparison of differentially enriched KEGG functions in CnmeGV-infected and non-infected groups at 24, 28 and 72 hpi. The enriched KEGG level 2 functions are shown in Supplementary Figure S7. Differences are considered significant at *p* < 0.05 using Welsh’s *t*-test and corrected with Benjamini–Hochberg FDR. The *p*-value, 95% confidence intervals and difference in mean proportions are shown in Supplementary Figure S7.

Infection of CnmeGV had a notable effect on the functional bacteria genes. There were substantial differences in KEGG orthologous (KO) pathways among all the groups according to ANOSIM analyses (*R* = 1, *p* = 0.001. Figure 8A). The PCoA results demonstrate the KEGG modules were clearly separated between CnmeGV-infected and mock-infected groups at the same hpi. There was a large overlap in the CnmeGV-infected groups between 24 and 48 hpi and a small overlap between the CnmeGV-infected group at 72 hpi and the mock-infected group at 48 hpi (Figure 8B).

A series of gut microbiota KEGG pathways changed after CnmeGV infection. The metabolism-related functions were enriched, but the organismal system-related functions were decreased at 24, 48 and 72 hpi (Figure 8C). Furthermore, based on level 2, the numbers of significantly changed KEGG pathways at 24, 48 and 72 hpi were 28, 17 and 26, respectively. The metabolism pathways of carbohydrates, amino acids, cofactors and vitamins were all higher in the CnmeGV-infected group at 24 and 72 hpi, and energy metabolism was higher at 48 hpi. In contrast, signal transduction and endocrine system were lower at 24, 48 and 72 hpi. There were higher antimicrobial resistance and lower immune systems in the CnmeGV-infected group at 24 and 72 hpi (Supplementary Figure S7).

## 4. Discussion

The roles of gut microbiota in the biological processes of host insects and the dynamic changes in gut microbe composition have attracted immense attention from researchers. Though diet and environment are considered to be the main factors in the formation of gut microbiota in insects, the interactions between gut microbiota loads and the pathogenicity of infectious pathogens also suggest an important role of pathogen infection in shaping the composition of the gut microbiota [24,25,47]. In this study, we performed a metagenomic analysis of the gut microbiota composition of *C. medinalis* larvae, which were infected by baculovirus CnmeGV, at the initial infection stage. Following the infection by a virus pathogen after invasion, the abundance and diversity of gut microbial communication changed dynamically. The compositional development of the gut microbiota in the CnmeGV-treated group was not consistent with the mock-infected group, thereby demonstrating that the gut profiles were driven by pathogen infection of CnmeGV. CnmeGV infection led to a change in the gut microbial abundance, and the numbers of all the OTU counts of the CnmeGV-infected group were relatively higher at the early and later stages than in the mock-infected group. This result was similar to another study; the culturable bacterial load was increased 18.2-fold in the gut of *S. exigua* after being infected with baculovirus SeMNPV [24]. It was also suggested that virus pathogen invasion caused the change in the bacterial diversity of gut microbiota. Bacterial species diversity in the healthy silkworm gut was greater when compared with the BmCPV- or BmBDV-infected silkworm gut [25,26]. Compared to the control, CnmeGV infection caused microbial diversity to drop sharply at the late stage (48 hpi and 72 hpi) but significantly increased at the early stage (24 hpi). The Chao 1, Shannon and Simpson diversity indexes considerably declined at 48 hpi and 72 hpi, further indicating a lower gut microbiota α-diversity following CnmeGV infection.

Firmicutes were the dominant bacteria in the gut of *C. medinalis* and were independent of the diet changes [28]. Though the larvae of *C. medinalis* infected with the virus still hosted Firmicutes as dominant bacteria, the abundance of Firmicutes was higher than in mock-infected larvae. Earlier studies also indicated that the proportions of Proteobacteria, which were dominant bacteria in two honeybee species, *Apis mellifera* Linnaeus, 1758 (Hymenoptera: Apidae) and *Apis cerana* Fabricius, 1793 (Hymenoptera: Apidae), increased markedly after infection with the Sacbrood virus [48]. The *Enterococcus* and *Staphylococcus* belonged to the dominant bacteria in *Bombyx mori* Linnaeus, 1758 (Lepidoptera, Bombycidae), and the abundance of both was increased after infection with BmCPV [25]. There was a predictable pattern in the greatly enriched and depleted taxa which were divided into four groups. *Enterococcus*, the domain bacteria in the gut of *C. medinalis*, was the most enriched bacteria with no particular association with different treatment groups. The abundance of the domain bacteria was important to gut microbiota homeostasis [49]. *Wolbachia* was also the core gut bacteria of *C. medinalis* and more highly enriched at 24 hpi and 72 hpi in the mock-infected group but at 48 hpi in the CnmeGV-infected group. *Wolbachia* has been discovered to protect *Drosophila* and mosquito species against RNA viruses [50,51] but had little impact on DNA virus infection [52]. *Wolbachia* was reported to enhance the susceptibility of *Spodoptera exempta* Walker, 1856 (Lepidoptera: Noctuidae) to baculovirus SpexNPV [53]. The different enrichment of *Wolbachia* in *C. medinalis* after CnmGV infection alerted us to further examination of the relationship between *Wolbachia* and DNA virus infection. Fifteen species were ultimately predicted as the biomarkers of CnmeGV infection, further confirming that CnmeGV infection changed the abundance of specific bacteria. Some species, including *Pseudomonas fluorescens*, *Stenotrophomonas maltophilia*, and *Xenorhabdus nematophila*, were pathogenic or opportunistic pathogens [54,55,56].

Moreover, changes in microbiota composition can affect the functioning of an ecosystem [57,58]. We also noticed that the infection of CnmeGV had a noteworthy effect on the functional bacteria genes. The genes involved in metabolism, including carbohydrate, amino acid, cofactor, and vitamin metabolism pathways, were significantly enriched after CnmeGV infection. Gut microbiota plays a pivotal role in providing nutrients and energy for the host [12,59,60]. Viral infections induced the Warburg effect, aerobic glycolysis, fatty acid synthesis and glutaminolysis, which increase energy and substrate availability for the viral cycle [61]. Gut bacteria encoded a series of metabolism-related enzymes involved in carbohydrate metabolism and amino acid synthesis, according to our previous studies [28]. The enrichment of metabolism function therefore could supply more nutrients and energy to maintain insect survival and the multiplication of the virus.

Gut microbiota is an active regulator of several aspects of host physiology, including the protective function of the host immune system, while the host immune system is an important factor in shaping the gut microbiota community [62]. The dynamic changes of gut bacteria were connected with host insect’s innate immunity [26]. Baculovirus SeMNPV infection led to a decreased expression of immune-related genes in a *Spodoptera exigua* Hübner, 1808 (Lepidoptera: Noctuidae) cell culture as well as in the larval gut, but the gut microbial loads were found to increase after virus infection [24]. Other studies have also suggested the that an imbalance of gut bacteria may suppress antiviral immune reactions, such as the suppression of prophenoloxidase expression to facilitate baculovirus infection [16,31]. In the present study, we found the disrupted functional bacteria genes associated with higher antimicrobial resistance pathways and lower immune system pathways in the CnmeGV-infected group at 24 and 72 hpi. CnmeGV infection induced a systemic antiviral response of *C. medinalis*, including the RNAi and oxidative stress antiviral mechanisms in our previous study [31]. These observations supported the scenario that CnmeGV infection triggered immune system activation in the gut and induced an enrichment of abundance and diversity of microbiota at an early stage. The change in gut microbiota disrupted the balance of the gut bacteria, which in turn activated anti-bacterial pathways, such as the oxidative stress and NF-κB pathways [16,31], to limit the further over-proliferation of gut bacteria, and caused the local extinction of numerous species. The rebalanced gut microbiota then interacted with the gut and negatively adjusted the immune response by reducing the level of reactive oxygen species (ROS), which were also harmful to the gut cells [63]. This, in return, worked to facilitate the virus infection and transmission indirectly [10].

## 5. Conclusions

In conclusion, the abundance and diversity of gut microbial communication of *C. medinalis* changed dynamically after the invasion of CnmeGV; according to the present analysis, the gut profiles of the host were driven by baculovirus infection of CnmeGV. Infection of CnmeGV also had a significant effect on the functional bacteria genes, including metabolism pathways and immune system pathways. Coupled with recent research, the interactions between baculovirus infection and gut profile changes were discussed. Further studies are required to confirm the connection between virus pathogenesis and gut microbe changes. Our study provided preliminary evidence for associations between specific gut microbiota features and CnmeGV infection, which might help unveil the mechanism of the interaction between microbiota loads and baculovirus infection.

## Data Availability

The metagenomic sequencing data used were deposited in the NCBI Sequence Read Archive under accession number PRJNA805352.

## Supplementary Materials

The following supporting information can be downloaded at: https://www.mdpi.com/article/10.3390/microorganisms12040757/s1. Supplementary Table S1: Numerical data of the gut microbiota metagenome of *C. medinalis*. Supplementary Table S2: Sequence number of different species in the *C. medinalis* gut. Supplementary Table S3: Sequence number of OTU classified as virus. Supplementary Figure S1: Rarefaction curves of the observed OTUs in different samples. Supplementary Figure S2: Box plots of frequency for the gut microbiota at the phylum level. Supplementary Figure S3: Krona plots display the classification and composition changes of bacteria in the larvae gut of *C. medinalis* after CnmeGV infection. Supplementary Figure S4: LDA score plot from LEfSe analysis shows the microbial taxa with significant differences between the CnmeGV-infected group (blue) and mock-infected group (red) (LDA score > 2). Supplementary Figure S5: Cladogram plot showing the taxonomic groups that explain the most variation among the bacterial communities. Supplementary Figure S6: ROC curve displaying the discriminatory potential of the random forest model in distinguishing CnmeGV-infection. AUC, area under curve. Supplementary Figure S7: KEGG pathways found at significantly different abundances in the gut microbiota metagenomics profiles as identified by PICRUSt and STAMP analysis.
