# Supplementary material for: Metagenomic Analyses Reveal Gut Microbial Profiles of Cnaphalocrocis medinalis Driven by the Infection of Baculovirus CnmeGV
Source: Microorganisms. 2024 Apr 9;12(4):757. doi: 10.3390/microorganisms12040757 (PMC11052019; doi:10.3390/microorganisms12040757)
Supplement: Supplementary file 1 [file microorganisms-12-00757-s001.zip › microorganisms-2901307-supplementary.pdf]

Supplementary Table S1 Numerical data of the gut microbiota metagenome of *C. medinalis*

Sample ID	Insert Size (bp)	RawReads (#)	RawBase (GB)	% GC	Clean reads (#)	Cleaned (%)	Clean Q20 (%)	Clean Q30 (%)
CnmeGV24	350	26739451	8.02	39	25268233	94.5	98.97	95.79
CnmeGV48	350	26222897	7.87	39	24887590	94.91	98.91	95.59
CnmeGV72	350	26712575	8.01	38	25358657	94.93	98.95	95.69
control24	350	26822439	8.05	38	25461569	94.93	98.97	95.77
control48	350	26956893	8.09	38	25536954	94.73	98.95	95.7
control72	350	28806158	8.64	38	27244287	94.58	98.96	95.76

Sample ID: sample name; InsertSize (bp): represents the use of a 350 bp Library; RawReads: sequencing number of raw reads; RawBase: total base pair number in the sequencing raw data, calculated by the product of RawReads and the sequence length; %GC: the percentage of GC content in the bases; Clean reads: the valid reads number after filtered; Cleaned (%): the percentage of Cleanreads to Rawreads. Clean Q20, Q30: the percentage of base numbers in CleanData with a mass value greater than 20 and 30.

Supplementary Table S2 Sequence number of different species in the *C. medinalis* gut

	Bacteria	Fungi	Archaea	Viruses
Sequence Number	6018759	28906	38	111

Supplementary Table S3 Sequence number of OTU classified as virus

#OTU ID	CnmeGV24	CnmeGV48	CnmeGV72	control24	control48	control72
Taxon00149	49	57	5	0	0	0
taxonomy						
k__Viruses;p__;c__;o__;f__Baculoviridae;g__Betabaculovirus;s__Cnaphalocrocis_medinalis_granulovirus						

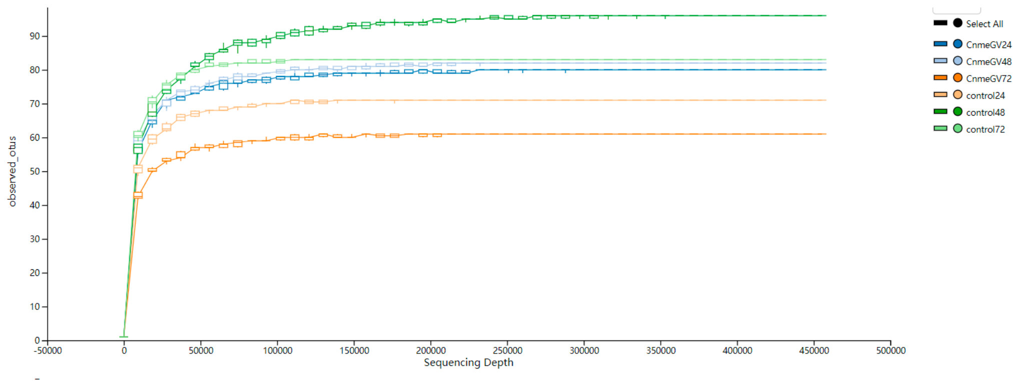

Supplementary FIGURE S1 Rarefaction curves of the observed OTUs in different samples.

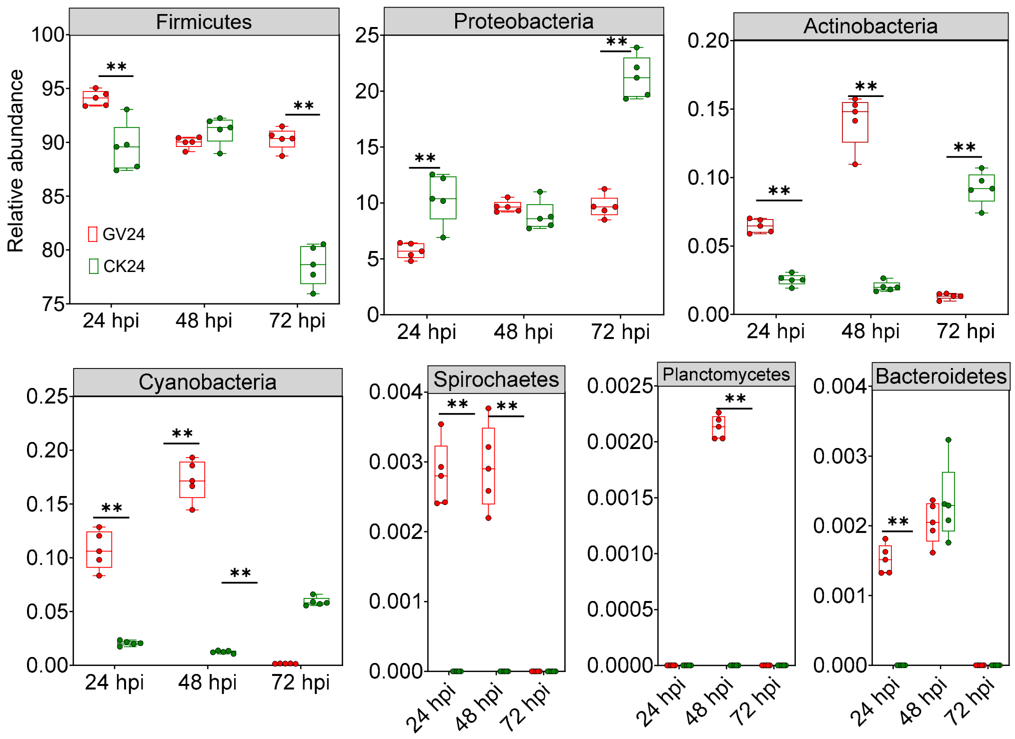

Supplementary FIGURE S2 Box plots of frequency for the gut microbiota at the phylum level

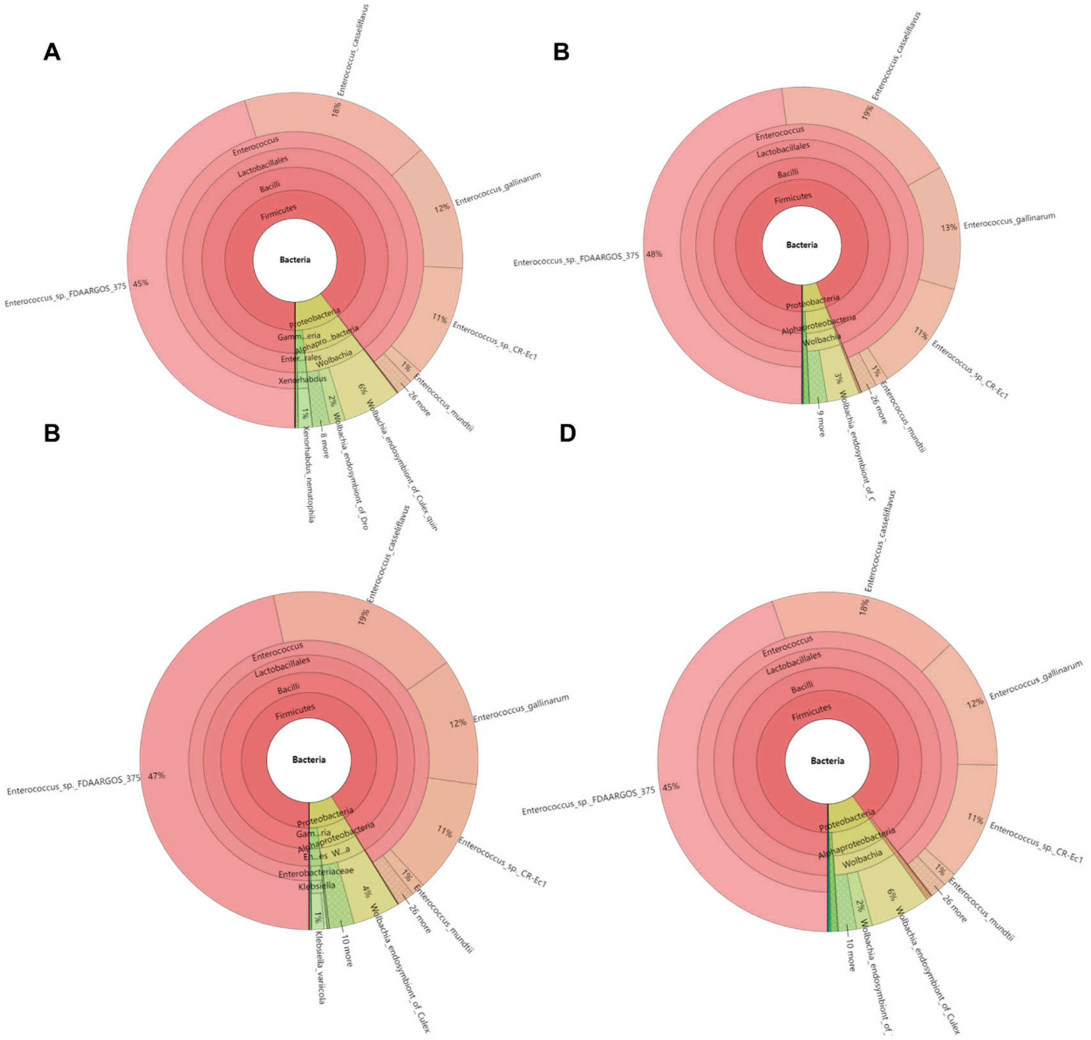

Supplementary FIGURE S3 Krona plots display the classification and composition changes of bacteria in the larvae gut of *C. medinalis* after CnmeGV infection. (A) Mock-infected at 24 hpi, (B) CnmeGV-infected at 24 hpi, (C) Mock-infected at 48 hpi, (D) CnmeGV-infected at 48 hpi.

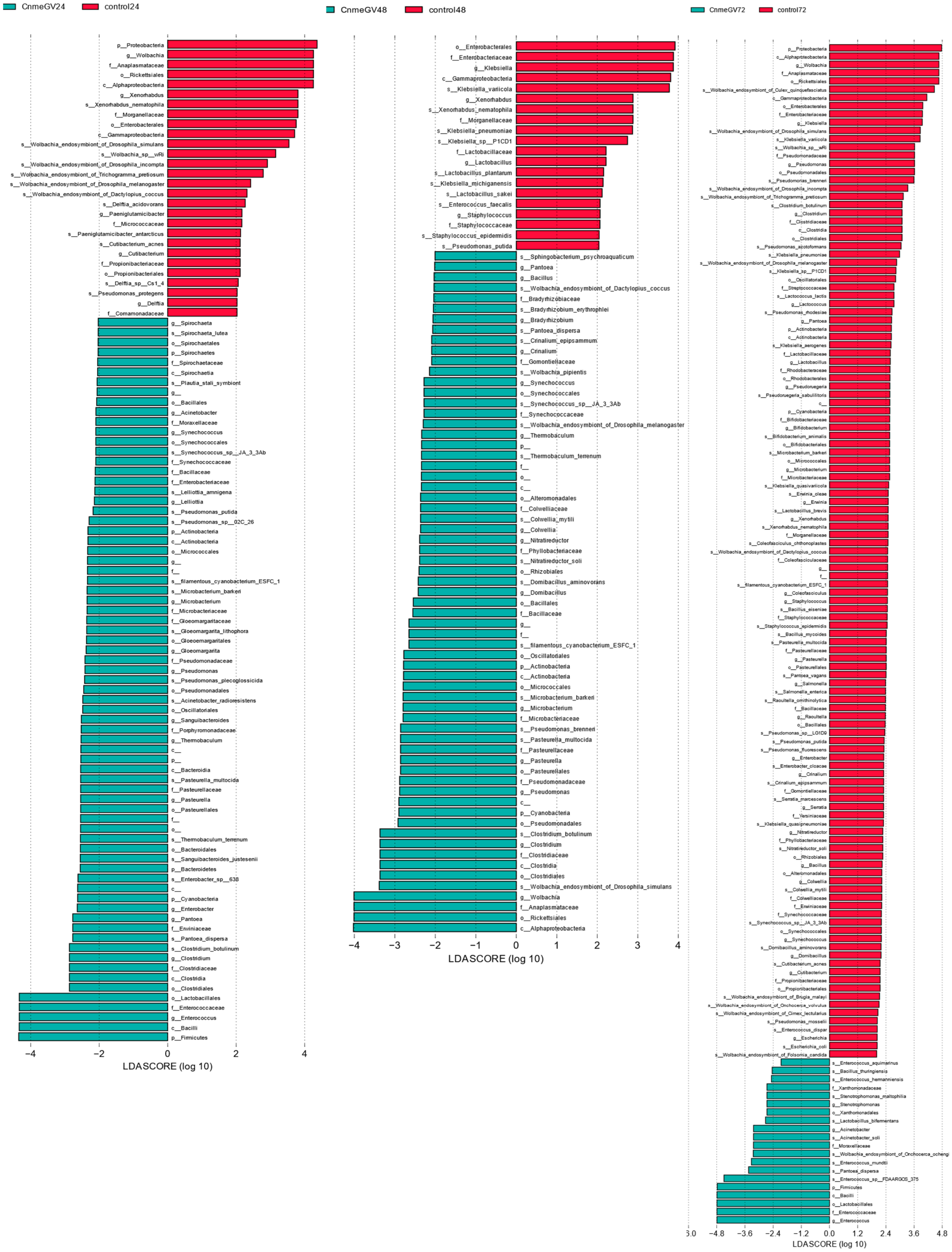

Supplementary FIGURE S4 LDA score plot from LEfSe analysis shows the microbial taxa with significant differences between the CnmeGV-infected group (blue) and mock-infected group (red) (LDA score > 2).

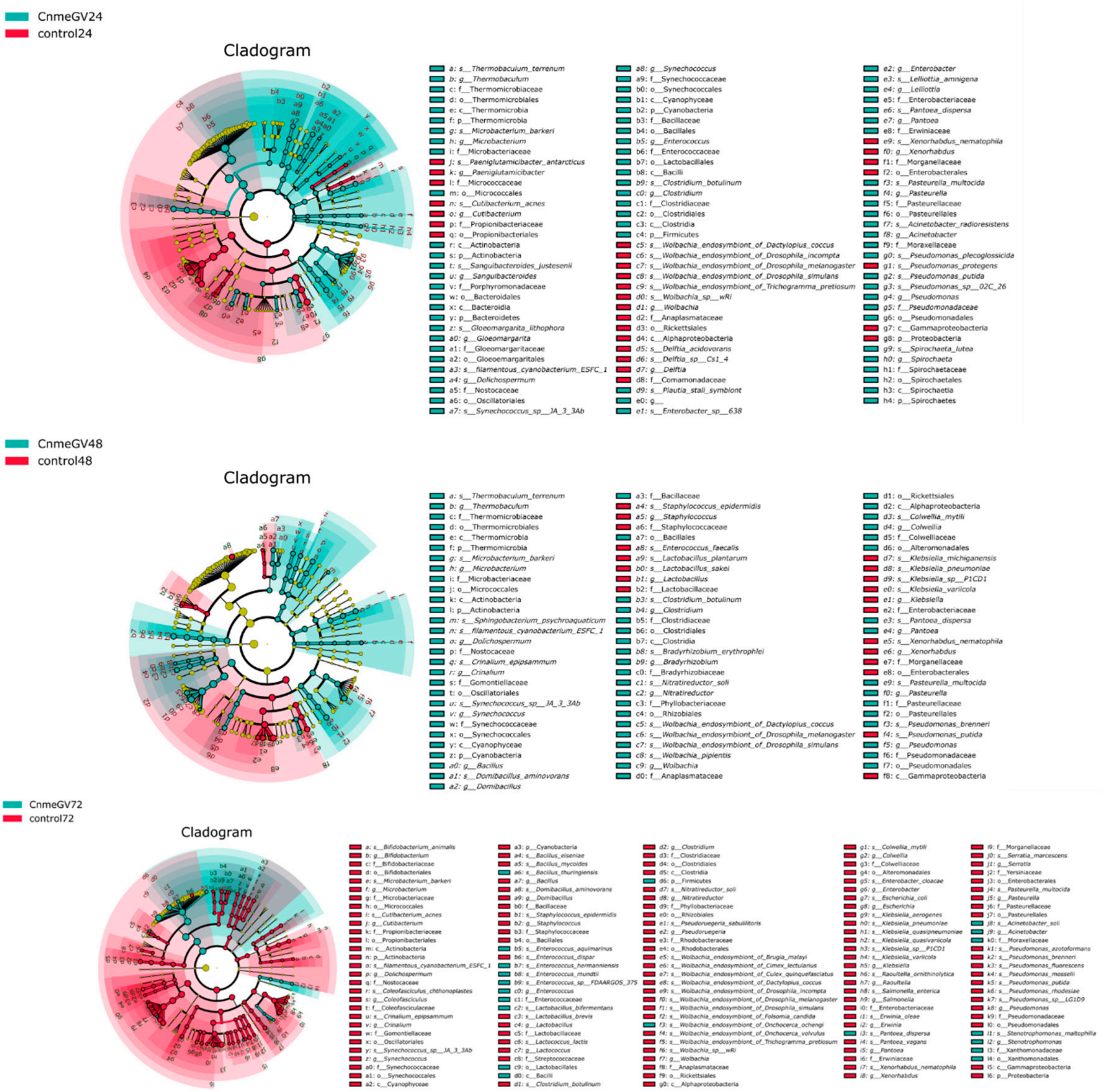

Supplementary FIGURE S5 Cladogram plot showing the taxonomic groups that explain the most variation among the bacterial communities. Differences are represented in the color of the significantly enriched class (red indicating mock-infected, blue indicating CnmeGV-infected, yellow non-significant).

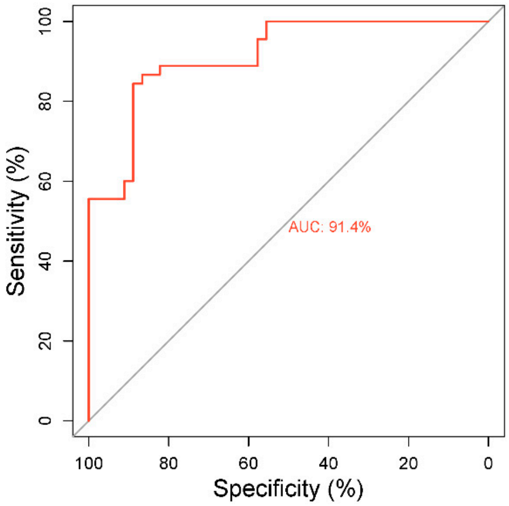

Supplementary FIGURE S6 ROC curve displaying the discriminatory potential of the random forest model in distinguishing CnmeGV-infection. AUC, area under curve.

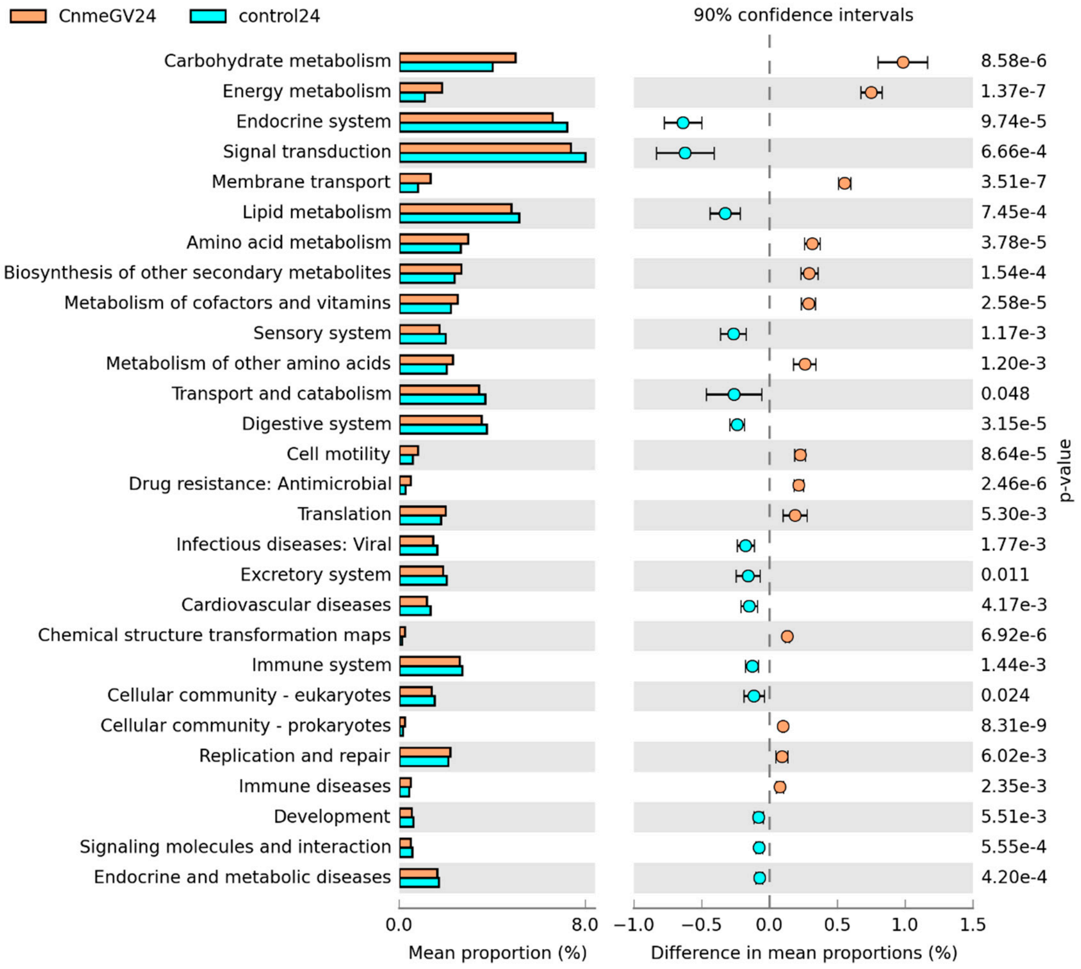

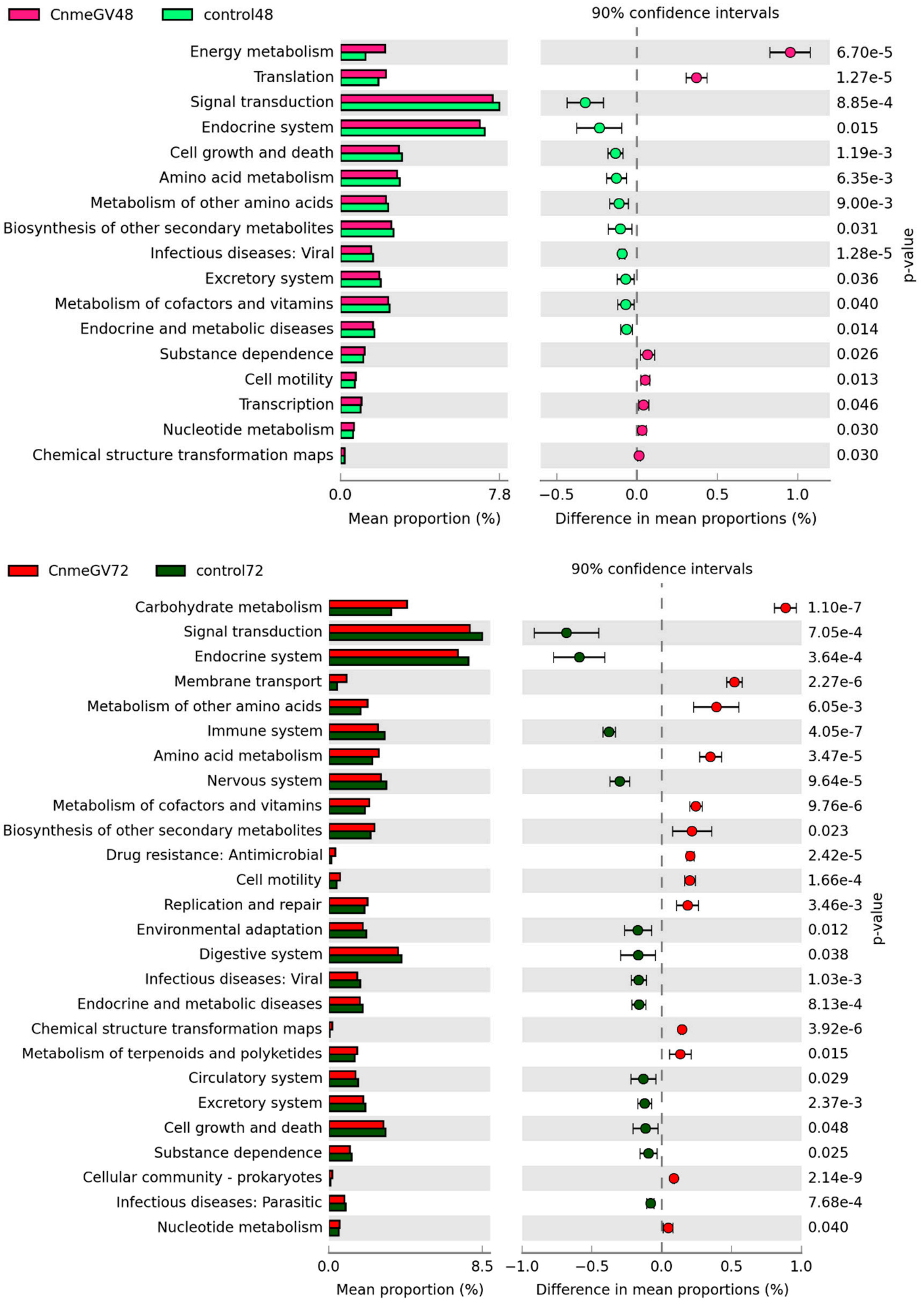

Supplementary FIGURE S7 KEGG pathways found at significantly different abundances in the gut microbiota metagenomics profiles as identified by PICRUSt and STAMP analysis.
